# Survey of radiation therapists’ current practices and perceptions of psychosocial and supportive care in Canada and Norway

**DOI:** 10.1007/s00520-025-09492-9

**Published:** 2025-05-05

**Authors:** Sophie Bezanson, Espen Aas, Kerry-Ann Smith, Lisa Di Prospero, Michael Velec, Arlinda Ruco, Safora Johansen

**Affiliations:** 1 Interdisciplinary Health Program, St. Francis Xavier University, Antigonish, Canada; 2 Department of Life Sciences and Health, Oslo Metropolitan University, Oslo, Norway; 3 Collaborative Academic Practice, University Health Network, Toronto, Canada; 4 Practice-Based Research and Innovation, Sunnybrook Health Sciences Centre, Toronto, Canada; 5 Princess Margaret Cancer Centre, University Health Network, Toronto, Canada; 6 Department of Radiation Oncology, University of Toronto, Toronto, Canada; 7 Peter Gilgan Centre for Women’s Cancers, Women’s College Hospital, Toronto, Canada; 8 Beatrice Hunter Cancer Research Institute, Dalhousie University, Halifax, Canada; 9 Nova Scotia Health, Halifax, Canada; 10 VHA Home HealthCare, Toronto, Canada; 11 Department of Cancer Treatment, Oslo University Hospital, Oslo, Norway; 12 Health and Social Science Cluster, Singapore Institute of Technology, Singapore, Singapore

**Keywords:** Psychosocial oncology, Supportive care, Radiation therapists, Cancer

## Abstract

**Purpose:**

Many cancer patients undergoing radiation therapy report unmet psychosocial needs, which can negatively impact their treatment outcomes and quality of life. This study explored the current practices and perceptions of radiation therapists (RTs) practicing in Canada and Norway with respect to addressing the psychosocial and supportive care (PSSC) needs of their patients.

**Methods:**

A cross-sectional study was conducted using an online evidence-informed survey of RTs in Canada and Norway that focused on (1) demographics, (2) RTs’ confidence level and perceptions related to PSSC, and (3) RTs’ current practices related to PSSC. Descriptive statistics, chi-square tests, and Mann–Whitney *U* tests were used to summarize the sample and compare differences between countries.

**Results:**

A total of 210 RTs completed the survey (Canada,* n* = 79; Norway, *n* = 131). RTs in both countries identified PSSC as an important aspect of quality care. Canadian RTs expressed a greater desire to expand their scope of practice in PSSC (*p* = 0.001). Norwegian RTs reported spending more time providing PSSC (mean: 8.3 h vs. 3.8 h; *p* < 0.001) and were satisfied with their current capacity. Barriers common to both countries included a lack of training and time constraints. Canadian RTs additionally reported limited institutional support.

**Conclusion:**

Significant differences exist in the current practices and perceptions of RTs in Canada and Norway regarding PSSC delivery. However, Canadian and Norwegian RTs agree that engaging in PSSC ensures the best care for patients undergoing radiation therapy. With enhanced training, knowledge translation of resources, and institutional support, RTs can better address the PSSC needs of their patients.

## Introduction

Over 60% of cancer patients undergoing radiation therapy experience psychosocial distress and reduced quality of life during their treatment [1, 2]. It is concerning that many patients continue to report unmet psychosocial and supportive care (PSSC) needs [1, 3–6], as these can result in a range of negative outcomes, including treatment delays, decreased satisfaction with care, and increased use of healthcare resources [7]. This underscores the importance of dedicating greater effort to addressing PSSC needs in order to ensure the best outcomes for patients. As radiation therapy is either the primary or adjuvant treatment for approximately 50% of cancer patients in Canada and Norway [8, 9], radiation therapists (RTs) have a unique opportunity to deliver PSSC through their consistent interactions with patients during treatment [10].

According to the Canadian Association of Psychosocial Oncology [11], psychosocial care recognizes the whole person, with a focus on treating the social, psychological, emotional, spiritual, and functional aspects of cancer. It is a multidisciplinary specialty of cancer care that aims to help patients cope with cancer management [11, 12]. Psychosocial care is patient-centered but may also involve support directed at their family members, friends, and caregivers [12]. Complementarily, supportive care is a coordinated approach to managing the symptoms and side effects of cancer that aims to enhance the quality of life of patients [13]. Such care may include support with pain relief, exercise, or offering dietary advice [13].

Throughout the course of radiation therapy treatment, RTs are the consistent point of contact for patients and are responsible for providing quality care that is safe, effective, patient-centered, timely, efficient, and equitable [14, 15]. Prior research has indicated that RTs are well positioned to address PSSC needs given their ability to develop trusting therapeutic relationships with their patients through regular interactions [10, 16]. By building trusting relationships with patients, using screening tools to identify unmet needs, and delivering educational sessions, RTs have helped reduce anxiety and enhance the quality of care their patients receive [7]. Therefore, RTs play an important role in supporting patients through navigating the complexities of a cancer diagnosis and the challenges that accompany treatment [10]. Although delivering PSSC falls within the scope of practice of RTs [11, 15], variations in their knowledge, skills, and judgment have been previously reported [7, 10, 16, 17]. Moreover, RTs have identified barriers to effectively delivering PSSC such as a lack of private consultation spaces, prioritizing the technical aspects of radiation therapy, limited knowledge or capacity to provide additional care, and time constraints [10, 17, 18]. The extent to which RTs are engaged in PSSC across different healthcare systems has not been previously reported.

This study explored the current practices and perceptions of RTs practicing in Canada and Norway with respect to addressing the PSSC needs of cancer patients undergoing radiation therapy. Both Canada and Norway are high-income countries and have comparable healthcare systems with universal access to care [19, 20], making a comparative analysis suitable. Prior research has explored PSSC delivery among Canadian RTs [10, 17]. However, the scope of these studies has been limited to RTs working at a single institution and thus may not be representative of clinical practice more broadly. Moreover, no such research has been previously conducted with Norwegian RTs or compared the current practices and perceptions of RTs across different healthcare systems internationally. This study compared RTs’ perceptions related to PSSC in two countries, one where clinical practice guidelines for PSSC exist and one where there are currently no such guidelines available. By comparing the clinical practices in both countries, the present study aimed to address this knowledge gap and identify opportunities to improve PSSC delivery.

## Methods

This was an international cross-sectional study using an online survey of RTs in Canada and Norway to assess their current practices and perceptions related to PSSC.

### Participants and recruitment

All currently practicing RTs in Canada and Norway were eligible to participate in this study. A recruitment email was sent to radiation therapy department managers in Norway at the beginning of April 2024. The email described the purpose of the study, with instructions to distribute the online survey to all RTs within their department (estimated at a total of 450 RTs in departments across Norway). In Canada, recruitment emails were sent out through the Canadian Association of Medical Radiation Technologists (CAMRT) to all RTs who held membership with the organization and were included in its listserv (estimated at 1400 RTs). The emails contained an invitation to participate and a link to the online survey.

### Survey development and data collection

The survey was evidence-informed and adapted from Larsen et al. [10] and Elsner et al. [21]. It focused on three main categories: (1) demographics, (2) RTs’ confidence level and perceptions related to PSSC, and (3) RTs’ current practices related to PSSC. The survey included 20 closed and open-ended questions, which took approximately 10 minutes to complete. A variety of multiple-choice, select all that apply, and 5-point Likert scale questions were used to gather data and gauge the level of agreement and confidence level. Open-ended questions were used to obtain a better understanding of participants’ perspectives and experiences engaging in PSSC. The survey categories and related questions are presented in Table 1*.*

**Table 1:** Categories and related questions from the survey distributed to RTs in Canada and Norway

Category	Questions
**Demographics**	
	In which province/territory do you currently practice?*
	What is your age?*
	What is your gender?*
	Do you work full-time or part-time hours?*
	How many years of working experience as an RT do you have?*
	What is your highest level of education completed?*
	What is your primary position?*
	What percent of your work time is spent on direct patient contact?*
**Confidence level and perceptions related to PSSC**	
Please indicate your level of ***confidence*** for the following statements (from 1 “Not confident at all” to 5 “Completely confident”)*	
	Articulating what PSSC entails*
	Initiating a conversation with your patients about PSSC*
	Addressing the PSSC needs of your patients*
	Using screening tools to identify unmet PSSC needs of your patients*
	Initiating referrals to other care providers for PSSC needs of your patients*
	Educating patients around PSSC*
	Listing effective interventions for addressing PSSC needs of patients*
	Finding local relevant patient resources for PSSC*
Please indicate your level of ***agreement*** with the following statements (from 1 “Strongly disagree” to 5 “Strongly agree”)*	
	RTs have a role to play in addressing the PSSC needs of cancer patients undergoing radiation therapy treatment*
	Addressing the PSSC needs of cancer patients undergoing radiation therapy treatment is within your scope of practice*
	My training (i.e., education and other professional training) has prepared me to address the PSSC needs of my patients*
	I have enough training in advanced communication skills*
	I feel confident in addressing the PSSC needs of my patients*
	I want to expand my capacity to address the PSSC needs of my patients*
	I am more likely to initiate PSSC with my patients and their families rather than for them to initiate it with me*
	Addressing the PSSC needs of my patients can make a difference for the quality of care that they receive*
	Addressing the PSSC needs of my patients can make a difference for their overall quality of life*
	I have clarity around my role as an RT related to PSSC*
	There are sufficient time and resources to provide patients with PSSC in my practice*
	Engaging in PSSC allows me to work to my full scope of practice*
	I am interested in expanding my knowledge about PSSC*
**Current practices related to PSSC**	
*Time dedicated to providing PSSC*	
	How many hours per week do you estimate you spend giving PSSC to your patients, if any?*
*Interventions*	
Please indicate whether you have implemented any of the following interventions in your practice to address the PSSC needs of your patients (select all that apply)	
	Initiated conversations around PSSC needs
	Provided individual information and/or additional educational sessions
	Provided group information and/or additional group educational sessions
	Provided patients with procedural information, describing the procedure and what to expect
	Provided information on various community support programs or services for people with PSSC needs
	Used a screening tool to identify unmet PSSC needs of your patients
	Used a screening tool to identify if psychosocial referrals were required for your patients
	Coached patients in anxiety reduction strategies
	Administered cognitive behavioral therapy
	Provided motivational interviewing
	Initiated referrals to other care providers to address unmet PSSC needs of your patients
	Involved family members, friends, and/or caregivers in supporting the patient
*Barriers*	
Do you experience any barriers to providing PSSC to your patients (Yes/No)?* If Yes, please indicate the factors limiting your ability to provide PSSC to your patients (select all that apply)	
	Ethics
	Financial resources
	Communication skills
	Equipment and practical resources
	Interdisciplinary collaboration
	Culture and/or language barrier
	Patient cooperation
	Accessing to training
	Institutional support
	Other
What supports, if any, would help you to address the PSSC needs of your patients?	

*Denotes items that were mandatory in order to advance in the survey

The survey was initially developed in English and translated into Norwegian. The Norwegian translation was reviewed in collaboration with members of the Canadian research team to ensure a contextually accurate translation. Prior to distribution, the survey was piloted with six RTs in Canada and six RTs in Norway to ensure face validity, clarity, and relevance. Changes and revisions were made to the survey based on the feedback received. Data were collected anonymously using Qualtrics© (May 2024) in Canada and Nettskjema (Universitet i Oslo, May 2024) in Norway. The survey was open for 6 weeks, and reminder emails were sent out after the second and fourth weeks to maximize participation. Data collection concluded on May 17, 2024.

### Data analysis

Descriptive statistics were used to summarize the study sample and all relevant variables. The primary comparison for all questions was between Canadian and Norwegian RTs. As a 5-point Likert scale was used in the study, the data obtained was ordinal and not normally distributed. Therefore, nonparametric Mann–Whitney *U* tests were used to analyze differences in the medians of Likert scale questions. Chi-square tests were additionally used for comparative analyses of ordinal data. Statistical significance was reported with unadjusted *p*-values at < 0.05. All analyses were completed using SPSS (version 29.0.1). Surveys that were incomplete because responses to the mandatory survey items were not completed were excluded from analyses. This included questions about confidence level and perceptions and current practices related to PSSC, as these were key constructs that we were interested in comparing between RTs practicing in both countries.

## Results

In total, 238 surveys were returned with an estimated response rate of 8% in Canada (*n* = 106) and 29% in Norway (*n* = 132). There were 27 Canadian participants and 1 Norwegian participant who were excluded from data analysis, as they did not return a complete survey. The following results are based on the adjusted total of 210 responses.

### Demographics

The demographic and work characteristics of the study participants, stratified by their country of practice, are presented in Table 2. Canadian RTs were significantly older than the Norwegian RTs in this study (*p* = 0.002), and the majority of the participants were women, including 90% of the sample in Canada and 78% of the sample in Norway. Participants worked in roles other than those listed, such as clinical informatics, pre-treatment resource therapy, radiation therapy review, and research. A few participants (4% overall) worked in multiple roles. Although most RTs in both countries reported that ≥ 50% of their work time was spent on patient contact, it was significantly higher among Norwegian RTs (80% vs. 61% for Canadian RTs; *p* = 0.002).

**Table 2:** Demographic and work characteristics of participants stratified by country of practice

	**Canada** **(*n* = 79)** ***n* (%)**	**Norway** **(*n* = 131)** ***n* (%)**	**Total** **(*N* = 210)** ***n* (%)**	***p-*value**
**Age (years)**				
< 40	22 (27.8)	60 (45.8)	82 (39.0)	0.002
≥ 40	57 (72.2)	64 (48.9)	121 (57.6)	
Prefer not to say	0 (0.0)	7 (5.3)	7 (3.3)	
**Gender**				
Man	7 (8.9)	29 (22.1)	36 (17.1)	0.023
Woman	71 (89.9)	102 (77.9)	173 (82.4)	
Prefer not to say	1 (1.3)	0 (0.0)	1 (0.5)	
**Job type**				
Full-time	66 (83.5)	120 (91.6)	186 (88.6)	0.024
Part-time	9 (11.4)	11 (8.4)	20 (9.5)	
Others	4 (5.1)	0 (0.0)	4 (1.9)	
**Work experience (years)**				
≤ 5	4 (5.1)	35 (26.7)	39 (18.6)	< 0.001
6–15	23 (29.1)	38 (29.0)	61 (29.0)	
16–25	27 (34.2)	36 (27.5)	63 (30.0)	
> 25	25 (31.6)	22 (16.8)	47 (22.4)	
**Primary position**				
Pre-treatment CT simulation	5 (6.3)	16 (12.2)	21 (10.0)	< 0.001
Dosimetry	4 (5.1)	15 (11.5)	19 (9.0)	
Treatment therapist	41 (51.9)	87 (66.4)	128 (61.0)	
Educator	10 (12.7)	1 (0.8)	11 (5.2)	
Manager	4 (5.1)	7 (5.3)	11 (5.2)	
Others	15 (19.0)	5 (3.8)	20 (9.5)	
**Patient contact (% of work time)**				
< 50	31 (39.2)	26 (19.8)	57 (27.1)	0.002
≥ 50	48 (60.8)	105 (80.2)	153 (72.9)	

### Confidence level and perceptions related to PSSC

**Fig. 1:**
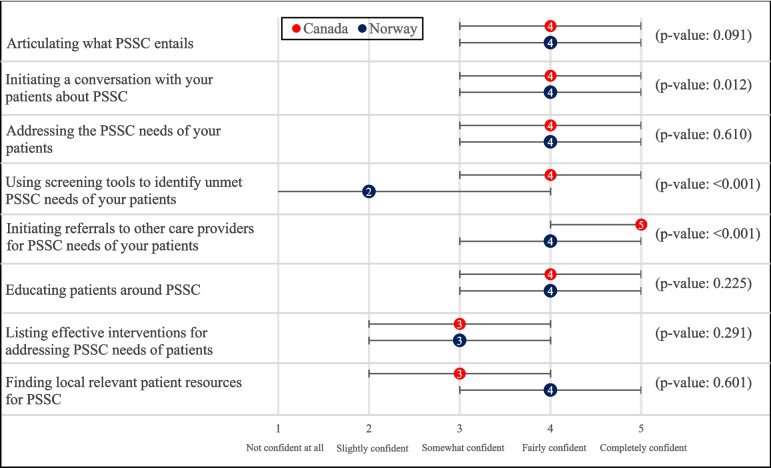
Median confidence levels, interquartile ranges, and *p*-values for statements related to RTs’ confidence level in addressing PSSC needs. The confidence level scale is displayed on the bottom axis

Canadian and Norwegian RTs were overall “Fairly confident” (median = 4) in addressing the PSSC needs of their patients, though some differences were noted with respect to certain tasks (Fig. 1). The significant differences in confidence level were that Canadian RTs expressed feeling “Completely confident” (median = 5) in initiating referrals to other care providers, while Norwegian RTs felt “Fairly confident” (median = 4) with such tasks (*p* < 0.001)*.* Moreover, Canadian RTs reported feeling “Fairly confident” (median = 4) using screening tools to identify unmet PSSC needs, whereas Norwegian RTs were “Slightly confident” (median = 2) in this area (*p* < 0.001).

**Fig. 2:**
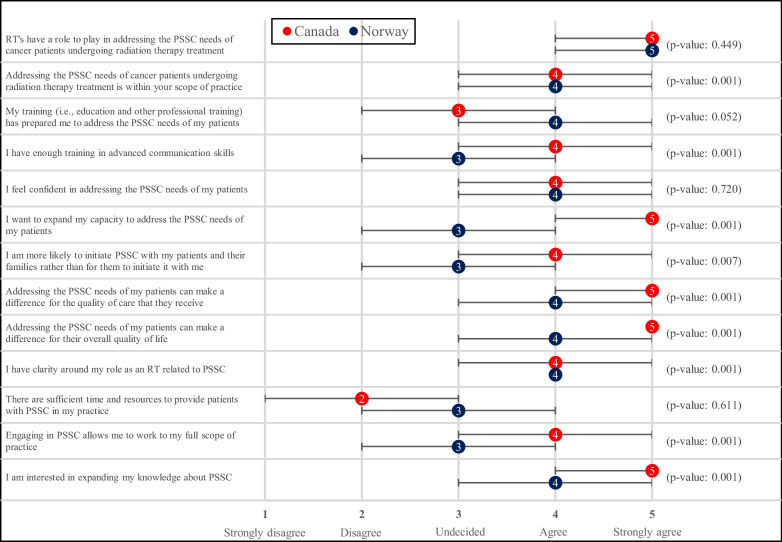
Median level of agreement, interquartile ranges, and *p*-values for statements related to RTs’ perceptions of PSSC. The agreement level scale is displayed on the bottom axis

In terms of perceptions, both Canadian and Norwegian RTs generally “Agreed” (median = 4) that RTs have a responsibility to address PSSC and that it falls within their scope of practice. However, there was a significant difference between Canadian and Norwegian RTs in their desire to broaden their capacity to engage in PSSC activities (Fig. 2). Canadian RTs expressed that they “Strongly agree” (median = 5) with an interest in expanding their capacity to deliver PSSC, while Norwegian RTs were “Undecided” (median = 3) about providing PSSC beyond their current capacity (*p* = 0.001). Canadian RTs additionally reported having significantly more training in advanced communication skills (*p* = 0.001) and interest in expanding their knowledge about PSSC (*p* = 0.001).

### Current practices related to PSSC

#### Time dedicated to providing PSSC

On average, Canadian RTs spent 3.8 h per week (SD 6.5) engaging in PSSC compared to Norwegian RTs who spent 8.3 h per week on PSSC activities (SD 8.7; *p* < 0.001). Canadian RTs commented that the role of RTs in providing PSSC has gradually decreased over time and has become increasingly focused on the technical aspects of care.

#### Interventions

**Fig. 3:**
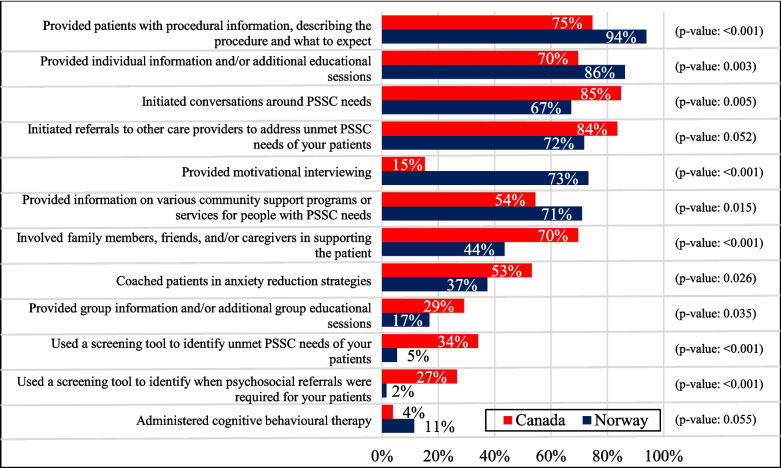
Current practice interventions among Canadian and Norwegian RTs utilized to effectively deliver PSSC

There were significant differences in the PSSC interventions used by RTs practicing in Canada and Norway. Canadian RTs were more likely to use screening tools to identify unmet PSSC needs (34% vs. 5% in Norway; *p* < 0.001) and involve family members and friends in supporting patients throughout their treatment (70% vs. 44% in Norway; *p* < 0.001) (Fig. 3). Conversely, Norwegian RTs facilitated motivational conversations more frequently (73% vs. 15% in Canada; *p* < 0.001). Most Norwegian RTs (94%) provided procedural information, whereas significantly fewer Canadian RTs (75%) provided such information (*p* < 0.001). In the open-ended questions, Canadian RTs commented that they often rely on quick conversations and digital surveys to assess patient needs. Norwegian RTs mentioned that they use individualized support to manage their patients’ anxiety and encourage physical activity, as well as provide patients with local resources to help them stay active and socialize.

#### Barriers

**Fig. 4:**
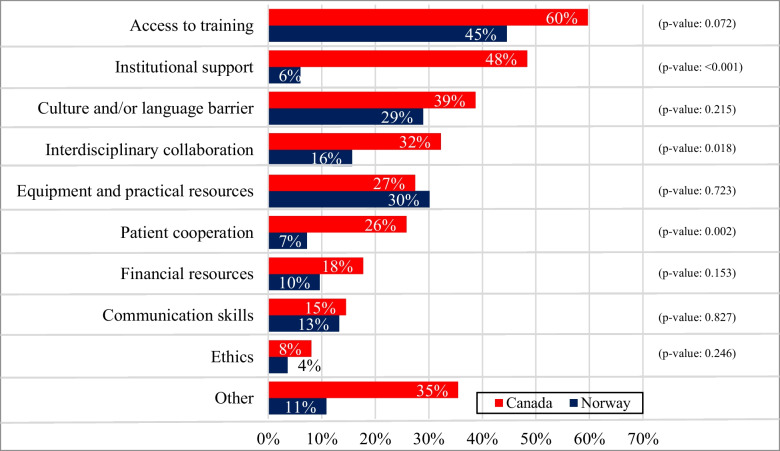
Barriers Canadian and Norwegian RTs reported facing when engaging in PSSC

There were 78% of Canadian RTs and 63% of Norwegian RTs who reported facing barriers to providing PSSC. The results presented in Fig. 4 are based on the RTs that have experienced barriers in addressing the PSSC needs of their patients. Both Norwegian and Canadian RTs frequently reported barriers related to access to training, with 60% of Canadian RTs and 45% of Norwegian RTs identifying this as a primary barrier. A significant difference was found in patient cooperation, with 26% of Canadian RTs reportedly experiencing issues in this area compared to 7% of Norwegian RTs (*p* < 0.001). Another significant difference was related to institutional support, in which 48% of Canadian RTs compared to 6% of Norwegian RTs reported a lack of such support (*p* < 0.001).

## Discussion

### Confidence level and perceptions related to PSSC

The results of this study regarding Canadian and Norwegian RTs’ perceptions related to PSSC align with previously published work. For example, Larsen et al. [10] previously noted that Canadian RTs identified PSSC as an integral aspect of providing quality care. Larsen et al. [10] further reported that engaging in PSSC enables RTs to gain greater personal fulfillment and satisfaction from their work. The level of fulfillment and satisfaction RTs derive from delivering PSSC may be shaped by their personal convictions and motivations, as some RTs with life experience or tragedy related to cancer were more motivated to provide such care [10]. Despite potential differences in personal convictions and motivations, many Canadian RTs in this study shared that providing PSSC allows them to fulfill their full scope of practice. Norwegian RTs identified PSSC as a key part of their responsibilities but were undecided about whether providing such care allowed them to work to their full scope of practice. These differences suggest that perceptions of PSSC are not only influenced by the personal convictions and motivations of RTs but may also be shaped by systemic factors or cultural conceptions of care within radiation therapy settings across both countries.

Despite similar confidence levels in addressing PSSC needs, Canadian RTs expressed a greater interest in expanding their knowledge and capacity to engage in PSSC compared to Norwegian RTs. Larsen et al. [10] previously revealed that Canadian RTs were interested in expanding their scope of practice to engage in PSSC by specializing in advanced practice radiation therapy or pursuing research in PSSC within their professional practice. Although Canadian RTs shared a greater desire to engage in PSSC, they reported feeling less equipped and prepared than Norwegian RTs to address the PSSC needs of their patients. This is an interesting finding given that expanding upon RTs’ current capacity to engage in PSSC requires sufficient time, resources, and support, which are currently limited in Canadian radiation therapy departments [22, 23]. Canadian RTs’ desire to expand their scope of practice within a healthcare system that is experiencing resource constraints, as well as workload and staffing pressures [22, 23], may reflect underlying cultural values about providing more holistic patient-centered care rather than simply addressing the physical aspects of disease. There is a growing focus on holistic patient-centered care in radiation therapy settings across Canada [10, 24], urging clinicians to consider the circle-of-care. This is reflected in the CAMRT Code of Ethics and Standards of Practice [25], as well as the clinical practice guidelines [11], which both stress the importance of RTs to focus on addressing the needs of both patients and their family members or caregivers in all aspects of their healthcare interactions.

Additionally, there may be important differences in professional practice, organizational structures, and institutional support around PSSC between the two countries. In Canada, the national clinical practice guidelines [11], as well as provincial resources for PSSC [26–28], may increase recognition of the importance of PSSC and contribute to the desire for an expanded scope of practice among Canadian RTs as seen in our study. Some Norwegian RTs indicated that their main focus was on the technical aspects of care and that delivering PSSC was the responsibility of other healthcare providers. There are no clinical practice guidelines for PSSC available in Norway that encourage RTs to integrate PSSC into their daily treatment, which may account for the differences noted between Canadian and Norwegian RTs with respect to their perceptions about which tasks fall within their scope of practice.

Canadian RTs in this study additionally reported a lack of clarity around their role in providing PSSC. Many Canadian RTs expressed a need for more PSSC resources and training, which is an interesting finding given that clinical practice guidelines for PSSC already exist as a resource in Canada [11]. This suggests that there may be a need for greater knowledge translation of these guidelines into clinical practice. Canadian RTs may also benefit from increased promotion of other complementary resources, such as provincial guidelines and resources [26–28], that will clarify and support their role in delivering PSSC. Norwegian RTs reported greater training and clarity regarding their role in addressing the PSSC needs of their patients, but were less interested than Canadian RTs in expanding their scope of practice. This is perhaps due to time constraints, as Norwegian RTs already spend significantly more time than Canadian RTs on PSSC delivery and may not be able to expand their current capacity. Moreover, Canadian RTs may be more interested in broadening their scope of practice, as some perceived a recent reduction in their time dedicated to providing PSSC and wish to return to delivering such care at their previous capacity. Even though perceptions related to PSSC differed between both countries, Canadian and Norwegian RTs agreed that addressing the PSSC needs of their patients is an important aspect of providing quality care.

### Current practices related to PSSC

In this study, Norwegian RTs estimated spending more time engaging in PSSC activities than Canadian RTs. Larsen et al. [10] previously reported that Canadian RTs spent 6.2 h per week on PSSC, which may indicate a reduction in providing such care over time. The time RTs dedicated to providing PSSC in the Larsen et al. [10] study was based on data collected from a single institution, whereas the present study encompassed RTs practicing across Canada. Despite these contextual differences, Canadian RTs in this study also shared that their time dedicated to providing PSSC has decreased over the last decade in response to an increasing demand for prioritizing the technical aspects of care. Canadian RTs have shifted focus to integrating PSSC into routine tasks and relying on screening tools due to time constraints [10, 17]. As there is no prior data from Norway, the results of this study suggest that Norwegian RTs dedicate more time on average to PSSC delivery. This may be a function of differences in organizational structures, as there are generally higher staffing levels in Norwegian radiation therapy departments compared to other countries [29], which may reduce individual workloads and allow RTs to dedicate more time to addressing the needs of patients.

This study further revealed differences in the types of PSSC practices utilized by RTs in Canada and Norway, which may reflect distinct healthcare approaches and systemic factors. Norwegian RTs emphasized the importance of personalized interactions with patients, which aligns with Newton et al. [30] who indicated that patients value direct personal support, and may account for the extended amount of time Norwegian RTs reported spending on PSSC activities. In contrast, Canadian RTs demonstrated a more structured approach to PSSC delivery, often involving family members or friends in PSSC and using screening tools to identify unmet PSSC needs. However, other studies reported limited effectiveness and support for the use of such tools to address patient needs [10, 17].

Most RTs in Canada and Norway reported experiencing barriers to effective PSSC delivery. Canadian RTs face ongoing systemic and organizational constraints, such as staffing shortages and inadequate funding, which may hinder their ability to provide comprehensive PSSC [17, 22, 23]. Hulley et al. [17] and Jones et al. [18] found that RTs are often constrained by high workloads and limited time, further restricting their capacity to effectively deliver PSSC. This aligns with barriers identified in our study, as RTs cited high workloads and time constraints as challenges that may contribute to the reduction in time spent on PSSC activities in Canada. These systemic pressures intensify staffing shortages and the fast-paced healthcare environments in which RTs work, which Sarra and Feuz [31] identified as contributors to burnout among RTs and reduced capacity for patient support.

Although Norwegian RTs benefit from greater institutional support, including higher staffing levels [29], they continue to face practical challenges in delivering PSSC, such as a lack of private spaces for patient interactions and time constraints. Merchant et al. [32] noted that radiation therapy environments that are not conducive to privacy can act as a barrier to communication between patients and RTs. Moreover, patients often pick up on time and efficiency pressures in radiation therapy departments, which can induce stress and anxiety during their appointments [32]. Effective communication and sufficient resources (i.e., time and space) are integral to reducing patient anxiety during treatment [33], which highlights a need for continued support and training that will enable RTs to deliver quality care. Elsner et al. [7] also noted that ongoing support and mentorship ensure the effective implementation of training into clinical practice. This underscores the need for enhanced training, resources, and institutional support in both countries to improve PSSC delivery and patient outcomes.

### Implications for clinical practice

The results of this study have several implications for clinical practice. Given the importance of PSSC for delivering quality care and maximizing patient outcomes, the role of advanced practice radiation therapists (APRTs) within the care team should be considered. APRTs can perform delegated tasks and effectively support patients in cancer management, as they possess additional knowledge and advanced clinical skills in a particular area of radiation therapy [34]. A feasibility study by Harnett et al. [34] revealed that APRTs already provide patient education, streamline referral processes, and ensure timely access to services in some radiation therapy settings in Canada. APRTs can choose to specialize in palliative radiation therapy and supportive care among other areas [34], although there is currently no option for specialization in PSSC. To this end, expanding professional practice for an APRT role specializing in PSSC would have the potential to mitigate time constraints and other institutional pressures.

As many RTs in our study were interested in expanding their scope of practice to engage in PSSC but may not have the capacity to become an APRT, it is important to leverage existing resources and tools that can improve the capabilities of all RTs with respect to delivering PSSC. For example, greater knowledge dissemination of the clinical practice guidelines in Canada [11] would ensure that RTs throughout the country are supported in delivering PSSC and more aware of how certain PSSC tasks may fall within their scope of practice. As there are no clinical practice guidelines for PSSC in Norway, it is recommended that such guidelines be created and disseminated to Norwegian RTs to encourage more widespread PSSC delivery and to hold RTs accountable to a national standard of care.

Both Canadian and Norwegian RTs reported time constraints as barriers to PSSC delivery, which could be addressed by integrating screening tools, such as the Edmonton Symptom Assessment Scale, more widely into practice or adopting new and efficient screening tools that have been implemented in comparable settings to identify patient needs. For example, the Patient-Reported Outcome Measures for Personalized Treatment and Care (PROMPT-Care) tool was recently introduced in Australia to improve screening efficiency for PSSC needs [16]. Although greater uptake among RTs is needed, with the appropriate education and training, this may prove to be a valuable tool that can help with referral processes, educating patients on radiation therapy, and identifying signs of anxiety, depression, and distress [16]. A similar tool could benefit RTs practicing in Canada and Norway if integrated into workflow, as it could help streamline processes for identifying patient needs and making referrals to other support services. Other strategies could include the investment in continuing professional development and additional training and education in advanced communication skills, which could support RTs in enhancing their ability to provide PSSC and feel more obliged to provide it [18].

### Strengths and limitations

This study addressed an important gap in the literature on PSSC, as it allowed for an international comparison of clinical practices between Canada and Norway, for which no prior comparison existed. An evidence-informed survey incorporating prior research on the topic was used to gain insight into the scope of PSSC delivery and opportunities for improvement. The survey was piloted with RTs practicing in Canada and Norway prior to the study start to ensure face validity. Moreover, participants were recruited from various Canadian provinces and Norwegian counties, which enhanced the external validity of the study findings within each country. However, this study also had some limitations. The low response rate from Canadian participants may have limited the generalizability of the study findings nationally. Additionally, recruitment in Canada was limited to RTs who received communication emails from CAMRT, and not every practicing RT is signed up for their listserv. This study also explored comparable populations of RTs, as both Canada and Norway are high-income countries with universal access to healthcare. However, this limits the external validity of the study findings, as results may not be generalizable to RTs working in low- or middle-income countries without universal access to healthcare. Future research should seek to explore if our results would be consistent in other such contexts. As there was no incentive provided to complete the study, participants may have primarily consisted of RTs who were most interested or experienced in delivering PSSC. The translation of survey questions from English to Norwegian may have additionally led to differences in interpretations of questions between participants in both countries. However, members of the Canadian and Norwegian study team collaborated on the survey development to ensure a contextually accurate translation and to mitigate this potential bias.

## Conclusions

This study explored the current practices and perceptions of RTs in Canada and Norway with respect to addressing the PSSC needs of cancer patients undergoing radiation therapy. Despite recognizing the importance of PSSC in providing quality care, RTs reported specific challenges related to PSSC delivery. Canadian RTs encountered limited institutional support, insufficient training in advanced communication skills, time constraints, and unclear role definitions, while Norwegian RTs expressed a lack of private spaces for patient interactions, limited access to specialized training, and heavy workloads as barriers. These issues may be addressed through targeted interventions, such as offering comprehensive training programs, enhancing institutional support by dedicating more time and resources to PSSC activities, clarifying RTs’ role in PSSC delivery, and investing in practical resources like private consultation spaces. Implementing these improvements can better equip RTs to address PSSC needs and aligns with a new model of healthcare reform that fully embraces the scope of practice of all members of the care team to enhance patient outcomes. Future research should employ in-depth interviews to gain a greater understanding of the barriers that RTs face in delivering PSSC in order to identify opportunities to maximize quality care in radiation therapy.

## Data Availability

No datasets were generated or analysed during the current study.
